# End-of-life care in the Dutch medical curricula

**DOI:** 10.1007/s40037-018-0447-4

**Published:** 2018-09-05

**Authors:** Josefien de Bruin, Mary-Joanne Verhoef, Joris P. J. Slaets, David van Bodegom

**Affiliations:** 1 Leyden Academy on Vitality and Ageing, Leiden, The Netherlands; 2 Centre of Expertise Palliative Care, Leiden University Medical Center, Leiden, The Netherlands; 3 University of Groningen, University Medical Center Groningen, Groningen, The Netherlands; 4 Department of Public Health and Primary Care, Leiden University Medical Center, Leiden, The Netherlands

**Keywords:** End-of-life care, Medical education, National blueprint

## Abstract

**Introduction:**

Future doctors must be trained in giving appropriate care to terminal patients. In several countries, medical curricula have been reviewed for the attention devoted to end-of-life care (ELC). In the Netherlands, no formal review had been performed. Therefore, the aim of this study was to provide an overview of the Dutch medical curricula regarding ELC.

**Methods:**

We formed a checklist based on international standards consisting of five domains of ELC education that are considered essential. Firstly, we studied the Dutch national blueprint on medical education. Secondly, using a questionnaire based on the checklist we studied the curricula of the eight medical faculties. A questionnaire was sent to all Dutch medical faculties to study the compulsory courses of the curricula. To assess the elective courses, we consulted the study guides.

**Results:**

The national blueprint included four of the five domains of ELC. None of the eight medical faculties taught all domains specifically on ELC; they were taught within other courses. Most attention was given to the domains on psychological, sociological, cultural and spiritual aspects; communication and conversational techniques; and juridical and ethical aspects. One faculty taught an elective course that included all essential aspects of the international standards.

**Discussion:**

Our study shows that ELC is currently insufficiently mentioned in the national blueprint and that none of the faculties fully integrated ELC as a part of their compulsory medical curricula. To improve ELC education, we recommend the Dutch Federation of University Medical Centres to add the five ELC domains to the national blueprint and we recommend the medical faculties to review their curricula and offer a separate and compulsory course on ELC to prepare their students for their future medical practice.

**Electronic supplementary material:**

The online version of this article (10.1007/s40037-018-0447-4) contains supplementary material, which is available to authorized users.

## Introduction

More people are living to ever increasing ages which has resulted in a large part of healthcare being devoted to chronic age-related diseases. Additionally, numerous diseases that used to be fatal have been turned into more chronic diseases by improved treatments. Medical and technical possibilities at the end of life have also increased substantially. All these developments have led to an increasing number of people needing complex end-of life care (ELC). To complicate matters, people also increasingly believe in the manipulability of the human life course and wish to be actively involved in decision-making. For the work of medical doctors, good ELC, as part of palliative care [1], is more important than ever before. At the moment, however, ELC is not yet optimal. For example, on the ICU, a third of the doctors questioned thought the care for at least one patient at that moment was disproportional, of which most care was considered futile and potentially harmful [2]. Moreover, doctors often offer palliative care too late and end-of-life discussions are not carried out frequently enough and often too late [3–5]. These factors influence the quality of life of terminal patients in a negative way [6, 7]. To improve the quality of life at the end of life doctors should be properly trained in ELC as a part of palliative care. Research has shown that students should be taught about ELC especially in the preclinical years of medical education, since these years are the most important for the development of basic skills, attitudes and knowledge for general medical practice. Besides, training in ELC does not conflict with other medical educational agendas since the acquired skills are useful to every healthcare specialization [8–10].

Worldwide, many studies have been performed to assess the attention to ELC in medical curricula. After having assessed the status quo of ELC in medical education, medical schools in several countries adapted their curricula to implement themes related to ELC, such as palliative care, hospice care and terminal care. In the United States, the first study that assessed the quality and quantity of ELC education was conducted in students and residents in 2003, concluding that fourth year medical students did not feel well-prepared to provide ELC and suggesting curricular changes and improvements in the medical working and educational sphere for students to learn how to provide good ELC [11]. In 2004, the same authors interviewed the deans of 51 medical schools in the US about ELC education in the curricula of their schools and concluded that most deans were willing to improve ELC education [12]. In Europe, the status of palliative care medical education in the undergraduate curricula of 43 countries was evaluated in 2015. Although palliative medicine was taught in a vast majority of European countries, there were substantial differences in the level of development of education about palliative medicine [13]. Several individual countries such as Switzerland, the United Kingdom and Germany studied their medical curricula and recommended curricular changes such as the addition of internships in hospices and more education about palliative care [14–17]. By contrast, little is known about the status of ELC in the Dutch medical curricula. In a report on appropriate care in the last phase of life, the Royal Dutch Association on the Advancement of Medicine (KNMG) stressed the importance of appropriate caregiving to terminally ill patients and of proper education about ELC [18]. However, no studies have been performed yet to determine to what extent ELC is taught in the Dutch medical curricula. Therefore, this study assesses ELC in the Dutch national blueprint (Raamplan Artsopleiding 2009) [19] and the Dutch medical curricula.

## Methods

To investigate to what extent ELC is currently taught at Dutch medical schools, we used the following definition of end-of-life, as described by the KNMG: ‘the phase of very old age, or the phase of a condition that will be life-threatening in the near future.’ Care for those who are at the end of life includes concepts such as appropriate care, curative and palliative care and over- or under-treatment [18].

To study the themes regarding ELC systematically, we designed a checklist. This checklist combined the criteria for essential elements in ELC education established by two international expert groups, [8, 20] and consisted of five main domains and twenty-two subdomains of ELC education (see Tab. 1).

**Table 1:** The five domains and 22 subdomains of essential ELC education composed of criteria formulated by Barnard et al. [7] and Emanuel et al. [17]

*Domain 1: Psychological, sociological, cultural and spiritual aspects*	Suffering
	Loss
	Mourning
	Rituals and meaning at the end of life
*Domain 2: Communication and conversational techniques*	Listening to the impact of disease on the patient’s life
	Explore hope, helplessness and fear in depth
	Discuss loss and mourning
	Discuss spiritual considerations
	Discuss advanced care planning
*Domain 3: Pathophysiology and treatment of symptoms*	Pain
	Dyspnoea
	Dehydration
	Depression
	Delirium
	Fear
*Domain 4: Juridical and ethical aspects*	Not-starting/stopping treatment and euthanasia
	Dilemmas on the treatment of pain
	Non-abandonment of the patient
*Domain 5: Self-reflection on personal and professional experiences with death and loss*	Personal experience with death
	View on the hereafter
	Goals of medicine
	Role of the doctor and other health workers in ELC

To assess the national blueprint and the medical curricula in the Netherlands, we took the same approach as a Dutch assessment of medical education on geriatrics: [21] we assessed the national level by studying the national blueprint and we assessed the faculty level by contacting the bachelor and master directors of medical curricula of the Dutch medical faculties.

Firstly, the national blueprint for higher medical education was studied using our checklist [19]. The national blueprint serves to secure that future doctors are trained in the basic competencies they need in their medical practice. This was done by two researchers (JdB en MV) independently. If they came to different assessments, their findings were discussed until agreement was reached.

Secondly, we assessed the curricula of the eight medical faculties by developing a questionnaire based on our checklist. The questionnaire studied the content and the didactic form of the current formal ELC education at the Dutch medical faculties. All eight medical schools in the Netherlands were approached: University of Groningen, University of Amsterdam, VU University Amsterdam, Leiden University, Erasmus University Rotterdam, Utrecht University, Maastricht University and Radboud University Nijmegen. To investigate the formal medical curricula, the coordinators of the bachelor and master programs of each medical faculty were invited to participate in the study. They could either submit the questionnaire by email or were interviewed (telephone interviews) using the questionnaire, which was done in almost all cases. In addition, the elective courses of each medical faculty were studied using online course catalogues or by contacting coordinators of the elective programs when course catalogues were not available online.

The study was carried out in accordance with the Declaration of Helsinki, including, but not limited to, there being no potential harm to participants, that the anonymity of participants was guaranteed, and that informed consent of participants was obtained. The study was approved by the institutional scientific review board.

## Results

### The national blueprint

The assessment of the national blueprint is described in Tab. 2. We found that four of the five domains were present in the national blueprint. The national blueprint did not mention the first domain on psychological, sociological, cultural and spiritual aspects of death and dying. The fourth and fifth domain were only marginally mentioned in the light of other subjects in the national blueprint.

**Table 2:** The presence of the five domains of end-of-life care (ELC) in the Dutch national blueprint for medical education

	End terms national blueprint
*Domain 1* Psychological, sociological, cultural and spiritual aspects of death and dying	–
*Domain 2* Communication and conversational techniques regarding ELC	6.2.2.4. After the bad news conversation, the young doctor is able to guide and support the patient and his loved ones adequately. 6.2.2.4. To guide and support the chronically and the terminally ill in palliative care
*Domain 3* Pathophysiology and treatment of symptoms in ELC	7.2.4.5. After the master program, the young doctor has knowledge about care for the terminally ill and the young doctor has knowledge about the dying process and about determining the cause of death. 8.3.1.2.4. After the bachelor program, the student has knowledge on the conception and development, growth and sexual maturation, and ageing and dying of an organism. 9.2.2.5. After the master program, the young doctor has knowledge on and insight in the conception, development, growth, sex maturation, ageing and dying process of an organism. 9.2.2.7. After the master program, the young doctor has knowledge on and insight in pathophysiology of the dying process and death of an organism
*Domain 4* Juridical and ethical aspects regarding ELC	*9*.3.3.10. After the master program, the young doctor has knowledge on and insight in the principles of medical ethics and can deal with dilemmas such as induced abortion or euthanasia
*Domain 5* Self-reflection on personal and professional experiences with death and loss	6.2.7.4. After the master program, the young doctor should have the competence to reflect on his/her own performance in difficult or moving situations. *6*.2.7.4. After the master program, the young doctor should have the competence to recognize his/her own feelings and norms and values in relation to existential questions on life, death, disease and health

### The medical curricula

The questionnaire for the assessment of the curricula was completed by all eight Dutch medical faculties (see Tab. 1 of the online Electronic Supplementary Material for the respondents and their positions). In five of the eight participating faculties, both the bachelor and the master education directors participated. In the case of Maastricht University, data were collected via another curriculum expert with permission of the bachelor and master coordinators. Since the respondent of Radboud University Nijmegen was responsible for both the bachelor and the master curriculum, a distinction could not be made. Data of the master program of Erasmus University Rotterdam were not available due to time restraints of the responsible persons.

Tab. 3 shows the assessment of the medical faculties. We found that none of the medical curricula taught all subdomains specifically on ELC and that no faculty addressed all five ELC domains in a specific course in the compulsory curricula. The form of ELC education varied. Education was considered specific on ELC if education was dedicated to the topic, for instance working groups on breaking bad news. Our study shows that 6 of the 8 faculties offered ELC-specific education for at least 1 subject. Of the 8 faculties, 2 did not offer ELC-specific education. The first domain was taught best, being offered specifically on ELC in 6 of the 8 faculties. The fifth domain received the least attention in Dutch medical education: in 4 of the 8 faculties, as ELC-specific education was only offered in 2 of the 4 subdomains.

**Table 3:** Education on end-of-life care in the Dutch medical curricula

	1		2		3		4		5		6		7		8
	B	M	B	M	B	M	B	M	B	M	B	M	B	M	B&M
*Domain 1: Psychological, sociological, cultural and spiritual aspects*															
Suffering	○	○	●	○	–	○	●	–	○	●	–	●	●	*n*/a	●
Loss	○	–	○	○	–	○	●	–	○	○	–	–	–	″	●
Mourning	○	–	○	○	–	○	●	–	○	○	–	–	+	″	●
Rituals and meaning at the end of life	–	–	●	●	–	○	–	–	–	–	●	–	+	″	–
*Domain 2: Communication and conversational techniques*															
Listening to the impact of disease on the patient’s life	○	○	○	○	○	○	●	○	○	○	●	●		″	●
Explore hope, helplessness and fear in depth	○	○	○	○	○	–	–	–	–	●	–	●	–	″	●
Discuss loss and mourning	○	–	○	○	○	–	●	–	–	○	●	–	–	″	●
Discuss spiritual considerations	–	–	○	–	–	–	–	–	–	–	–	●	–	″	●
Discuss advanced care planning	–	○	○	○	○	–	●	–	●	●	–	●	–	″	○
*Domain 3: Pathophysiology and treatment of symptoms*															
Pain	○	○	●	○	○	○	●	●	●	○	●	●	○	″	●
Dyspnoea	○	○	○	○	○	○	–	○	○	○	○	●	○	″	●
Dehydration	○	○	○	○	–	○	○	○	○	–	○	●	○	″	●
Depression	–	○	○	○	○	○	–	○	●	–	●	●	○	″	○
Delirium	–	○	○	○	–	○	●	○	○	○	○	–	○	″	○
Fear	–	○	○	○	○	○	–	○	●	–	●	–	○	″	○
*Domain 4: Juridical and ethical aspects*															
Not-starting/stopping treatment and euthanasia	○	○	●	●	○	○	●	○	●	○	●	●	●	″	–
Dilemmas on the treatment of pain	○	○	○	●	–	○	●	●	+	○	–	–	+	″	–
Non-abandonment of the patient	–	○	○	○	–	–	●	○	○	–	○	○	○	″	–
*Domain 5: Self-reflection on personal and professional experiences with death and loss*															
Personal experience with death	–	–	●	●	–	○	●	–	–	●	●	–	–	″	–
View on the hereafter	–	–	○	–	–	–	–	–	–	–	–	–	–	″	–
Goals of medicine	○	○	○	○	○	○	–	–	○	○	○	–	○	○	○
Role of doctors and other health workers in ELC	–	○	○	○	–	○	●	–	○	○	–	●	–	″	○

● Interactive, ELC-specific education; working group, discussion group, practical sessions, + Passive, ELC-specific education; lectures, ○ Non-ELC-specific education; interwoven in courses, – No ELC-education, *n*/*a* Not available, *1* University Medical Center Utrecht, *2* VU University Medical Center, *3* University Medical Center Groningen, *4* Leiden University Medical Center, *5* Amsterdam University Medical Center, *6* Maastricht University Medical Center, *7* Erasmus University Medical Center, *8* St. Radboud University Medical Center, *B* bachelor, *M* master, *n/a* not available

Most ELC-specific education was taught in an interactive way; only 2 faculties used passive educational forms (i.e., lectures) to educate their students about 4 subjects regarding ELC.

In all domains, ELC-related subjects were interwoven in lectures, working groups, discussion groups or practical training on more general topics; this education is not specific on ELC, but ELC does have a place in this education. For example, *treatment options for pain in ELC* was often part of a lecture on pain treatments in general. The University Medical Center Groningen facilitates education driven by students’ preferences and therefore does not offer ELC-specific education in the compulsory curriculum.

We also studied the elective curricula of the eight faculties. One faculty, Radboud University Nijmegen, offered the elective course *Coping with death* that covered all five domains. Three of the eight faculties taught in total five elective courses in which ELC plays a role: *Pain and pain treatment*; *Palliative care*; *Oncology; Paediatric oncology*; *Intensive care medicine,* and *Ethics in health care*.

## Discussion

The five domains of ELC that are considered essential are currently not taught to medical students of all faculties. This is an important observation when taking into account the growing attention to the patients’ quality of life at the end of life which demands proper training in ELC for all medical doctors.

### The national blueprint

We found that the national blueprint contained only four of the five domains of ELC education that are considered essential skills, knowledge and attitudes for young medical doctors. Furthermore, the domains are described in a very general way and can be easily overlooked in the national blueprint. Lack of national guidelines for ELC and palliative care has led to legal implementation of palliative medicine education in Germany and Switzerland and the national curricula on palliative care in Australia (the Palliative Care Curriculum for Undergraduates Initiative) [22] and Canada [23].

The new CanMeds Model of 2015, which serves as an international guideline for medical education around the world, holds several improvements in the light of ELC education. Most importantly, it now explicitly describes the key competence to discuss cultural matters, which includes beliefs about the end-of-life. However, the new CanMeds model still lacks several domains of the international checklist and we therefore recommend addition of all ELC domains.

### The medical curricula

The most prominent finding of the curricular assessment was that none of the eight faculties offered complete ELC education. This indicates that ELC education is not yet well-developed in the compulsory medical curricula in the Netherlands. These results are consistent with existing literature from other countries. One study, investigating the extent to which palliative medicine was taught in the Swiss medical curricula [14], showed that not all domains were covered in all curricula of the different medical faculties. Furthermore, a series of studies measured the status of palliative care education in the UK, indicating that at first, in 1983, only 4 of the 24 medical schools taught (informal) education dedicated to palliative care [24]. These findings led to opportunities to alter and test the medical curricula regarding ELC education, which is done regularly in the UK and Germany [16, 25, 26].

Currently, the attention of the ELC domains in the curricula varied. This is in line with a study on palliative care in medical education at a European level: in 27% of the countries, the faculties could determine whether and how they teach palliative care and consequentially the quality of palliative care education differed within these countries [13].

Only one faculty (Radboud University Nijmegen) offered an elective course that paid attention to all the domains. Here, we see opportunities to develop and share educational programs on ELC to improve the elective program.

There are numerous possible explanations for the shortage of attention to ELC in the medical curricula. Firstly, there is still a taboo on talking about death and dying [5]. Furthermore, since many practising physicians were never formally trained in ELC themselves, this makes it difficult to pass this knowledge on to future generations. Moreover, as mentioned by two respondents who acknowledged the importance of ELC in medical education, other subjects were prioritized over ELC because ELC was not described explicitly in the national blueprint. These and other reasons for incomplete ELC education are described extensively in the report on *Appropriate care in the last phase of life*, published by the Royal Dutch Association on the Advancement of Medicine [18].

This overview of current ELC education has several implications for practice. Firstly, since the national blueprint does not cover all the aspects of ELC education that are considered essential knowledge by international standards, we recommend to add all five domains and their subdomains to the national blueprint. The absence of the multi-dimensional approach of ELC in the national blueprint results in deprioritizing of ELC at the faculty level.

Secondly, this study suggests that exchange of information and knowledge on ELC education can improve Dutch medical education on ELC. For example, Radboud University Nijmegen developed an elective course on ELC that included all five domains of our checklist and this course could therefore serve as a model for other faculties. Thirdly, this study can be used to compare curricula with international medical education standards and to identify room for improvement. Fourthly, this study can be used as a baseline measurement for testing future curricular changes. And lastly, the established questionnaire for the different faculties can be used as a measurement tool for further research on ELC education in the future.

### Limitations

There are four possible limitations of this study. Firstly, the data of the master medicine of the Erasmus University Rotterdam were not available because of time restraints of the responsible persons. Therefore, our overview of ELC in Dutch medical education is not fully complete. However, since otherwise all the data were collected, this study still provides a reliable overview of the medical curricula and indicates many possibilities for improvement of medical education dedicated to ELC. Secondly, since ELC was mainly integrated into other compulsory courses, the education directors reported that it was difficult to give a precise indication of the presence and time spent on the domains on ELC. Therefore, they may have given a more positive or negative view of their curricula, which would make our assessment an overly optimistic or overly pessimistic view of the current situation of ELC education. Thirdly, since the curricula are always in development, this review provides a cross-sectional view that possibly contains parts of old and new curricula. Fourthly, some respondents reported that the questionnaire was difficult to fill in, because ELC education was often part of education about other topics. Therefore, we opted to discuss the results with the respondents using telephone interviews, which was done in almost all cases.

Although this research shows the current situation of ELC education in the Netherlands regarding the national blueprint and the curricula, no studies have been performed to assess the individual level of the skills and knowledge of the students. Further research should therefore focus on the personal experience and knowledge regarding ELC of the medical students themselves. Other international studies also studied to what extent future doctors feel prepared to provide ELC. This will give further indications on how to better prepare medical students for their future medical practice. At the time of writing this article, the PASEMECO project of Maastricht University is assessing students’ skills and knowledge regarding ELC and developing and implementing e‑learning on palliative care in the Dutch medical curricula.

## Conclusion

Our study shows that ELC is sparsely described in the Dutch blueprint for medical education: it is not explicitly mentioned as a compulsory subject and not all domains that are considered essential knowledge and skills by the international standards are represented in the national blueprint. This has consequences for the planning and execution of the medical curricula at the faculty level. First of all, ELC was part of the formal curricula, but none of the faculties taught all the subjects that were considered a necessary basis for ELC practice. Moreover, ELC was not offered as an individual course in any of the Dutch bachelor and master medical curricula. Secondly, none of the medical faculties taught the five domains on ELC and met international criteria. To improve ELC education, we recommend addition of all the ELC domains that are internationally accepted to the national blueprint. Besides, we recommend medical faculties to offer a separate compulsory course on ELC to educate and prepare their future doctors properly, so that people in an ageing world can rely on young medical doctors who feel ready and well-informed when providing appropriate ELC.

## Caption Electronic Supplementary Material

Supplementary Tab. 1. Characteristics of the respondents
