# Supplementary material for: End-of-life care in the Dutch medical curricula
Source: Perspect Med Educ. 2018 Sep 5;7(5):325–31. doi: 10.1007/s40037-018-0447-4 (PMC6191393; doi:10.1007/s40037-018-0447-4)
Supplement: Supplementary file 1 — Supplementary Tab. 1. Characteristics of the respondents [file 40037_2018_447_MOESM1_ESM.docx]

**Supplementary Table 1. Characteristics of the respondents.**

| Medical  faculties | Respondents | |  |
| --- | --- | --- | --- |
| UMCU | Director bachelor and master medicine | |  |
|  |  | |  |
| VUMC | Director bachelor medicine  Director master medicine | |  |
|  |  | |  |
| UMCG | Director bachelor medicine  Director master medicine | |  |
|  |  | |  |
| LUMC | Head of the Centre of Expertise Palliative Care + Coordinator Course Last Phase of Life  Director master medicine | |  |
|  |  | |  |
| AMC | Director bachelor medicine  Director master medicine + Coordinators Course Family Medicine and Course Medical Professional trainer | |  |
|  |  | |  |
| Erasmus MC | Interim Director bachelor medicine | |  |
|  |  | |  |
| MUMC | Researcher of the PASEMECO project (with consent of the bachelor and master coordinators) | |  |
|  |  | |  |
| RadboudUMC | Professor of Pain and Palliative Care | |  |
|  |  | |  |
|  | |  | |
| Abbreviations: UMCU: University Medical Center Utrecht; VUMC: Vrije Universiteit Medisch Centrum Amsterdam; UMCG: University Medical Center Groningen; LUMC: Leiden University Medical Center; AMC: Academic Medical Center (Amsterdam); Radboud UMC: Radboud University Medical Center (Nijmegen); Erasmus MC: Erasmus Medical Center (Rotterdam); MUMC: Maastricht University Medical Center. | | | |
